# Impact of living and training at low altitude for several years on endurance performance at higher altitude: Myth or reality

**DOI:** 10.1113/EP093863

**Published:** 2026-08-29

**Authors:** Fabienne Durand, Sergi Brau Villaro, Antoine Raberin

**Affiliations:** ^1^ Espace‐Dev UPVD Perpignan France; ^2^ Espace‐Dev IRD, Univ. Montpellier Montpellier France; ^3^ Institute of Sport Sciences University of Lausanne Lausanne Switzerland

**Keywords:** adaptations to exercise, altitude residence, endurance training, hypoxia, performance

## Abstract

The physiological adaptations of long‐term high‐altitude residents without high‐altitude genetic ancestry remain poorly characterized. This study investigated whether permanent residence at low altitude (1200 m) influences performance at higher altitude (2500 m). We compared exercise responses at 2500 m in endurance‐trained athletes living for several years at 1200 m versus those residing at sea level. Twenty‐five male endurance athletes [13 altitude residents (AR) and 12 sea‐level residents (SLR)] performed maximal incremental cycle ergometer tests in their habitual environment (BASE: 1200 m or sea level) and in simulated moderate altitude (2500 m, inspired $P_{O_{2}}$ = 109 mmHg). Gas‐exchange, heart rate, oxygen saturation, ventilatory and performance outcomes were measured continuously. At BASE, no between‐group differences were observed in anthropometrics, spirometry or aerobic capacity, while ventilatory reserve was more reduced in the AR group (*P =* 0.004). Both groups exhibited significant reductions in aerobic capacity, maximal oxygen saturation and peak power output at 2500 m compared with BASE. However, AR athletes exhibited a lower percentage drop in maximal oxygen saturation (*P <* 0.001) and aerobic capacity (*P =* 0.027). Maximal heart rate decreased significantly in SLR but remained unchanged in AR. At rest, the 2500 m condition significantly reduced oxygen saturation in SLR (*P <* 0.001) but not in AR. In conclusion, long‐term residence and training at 1200 m attenuate hypoxia‐induced performance decrements at 2500 m, mainly through better preservation of arterial oxygen saturation. Chronic exposure to low altitude might therefore induce functional pulmonary adaptations, with practical implications for endurance athletes training or competing at moderate altitude.

## Abstract (highlights)

- **What is the central question of this study?**
  Although responses to exercise at high altitude in resident populations have been well studied, less is known about long‐term residence at low altitude. Do living and training at low altitude for several years influence endurance performance at higher altitude?
- **What is the main finding and its importance?**
  Chronic residence at low altitude (∼1200 m) is associated with sustained ventilatory adaptations, including higher maximal oxygen saturation and significant depletion of ventilatory reserves during maximal exercise. Athletes residing at low altitude are able to maintain higher aerobic capacity and maximal power output during exercise at 2500 m, accompanied by higher values of maximal oxygen saturation, despite reduced ventilatory reserves.

## 1 INTRODUCTION

One of the most remarkable performances (climbing speed) achieved at extreme altitude was accomplished in 2017 by a renowned professional endurance athlete (Millet & Jornet, 2019). This athlete has dominated international endurance competitions in his main disciplines, trail running and ski mountaineering, for several years. To achieve these performances, this athlete has undergone full‐time, systematic endurance training since the age of 13 years (Jornet, 2017). Beyond his training load, his lifelong living environment must also be considered. He was born and raised in Cerdanya, a high plain with an average elevation of 1200 m (maximum summit: 2921 m), classified as low altitude according to Bärtsch and Saltin (2008). Throughout childhood and adolescence, his training was conducted exclusively within this environment, prior to one of his first major international achievements, becoming the youngest winner of the Ultra‐Trail du Mont Blanc in 2008.

There is evidence of the beneficial effects of altitude training to perform at a similar altitude (Fulco et al., 2000). Recently, Chapman et al. (2016) have shown that athletes who train to perform at altitude should live (acclimatize) at the target altitude and not higher. However, all included subjects of these studies are athletes who live at sea level and acclimatize for days to weeks, before performing at altitude. Therefore, these findings are not directly comparable to athletes who have resided permanently at low to moderate altitude. The case of K. Jornet should instead be considered that of a ‘lifelong acclimatized’ individual, who cannot be meaningfully compared to a sea‐level athlete participating in a short‐term altitude training camp.

Regarding lifelong and transgenerational adaptations, the physiological adaptations of Andean and Himalayan high‐altitude natives have been well documented (Beall, 2007; Getu et al., 2026; Gilbert‐Kawai et al., 2014). However, these ethnic groups [whose distinct genetic traits, including reduced alveolar–arterial oxygen difference, lower ventilation (${\dot{V}}_{E}$), higher haemoglobin mass and elevated oxygen saturation ($S_{pO_{2}}$), enable better maintenance of aerobic capacity (${\dot{V}}_{O_{2}\max}$) in hypoxia compared with Caucasian populations (Brutsaert, 2008)] reside at altitudes substantially higher than 1200–2200 m. Their adaptations are therefore not directly comparable to those of Caucasian individuals, whether lifelong residents of mid‐altitude plateaus or multigenerational inhabitants of such environments. The case of Kenyan runners, who come from the Kalenjin, an area located at an elevation ranging from 1830 to 2450 m (Pitsiladis et al., 2004), would therefore surely be more comparable. At altitudes comparable to those of Cerdanya (1200–2921 m), it has been reported that hypoxia is not the sole factor contributing to the success of these distance runners. Indeed, factors such as specific somatotypes promoting biomechanical and metabolic efficiency, strong motivation driven by the socioeconomic rewards of success in distance running, early initiation of consistent aerobic training, and moderate‐volume, high‐intensity training at altitude have been identified (Wilber & Pitsiladis, 2012).

Finally, only a few studies have focused on ‘lifelong acclimatized’ Caucasians with a European lifestyle in a classic socio‐economic environment. Within this context, previous studies have investigated the effects of residing at moderate altitude on the health outcomes of populations in Switzerland and Austria, yet without examining physiological adaptations to exercise in hypoxic conditions (Burtscher et al., 2021; Faeh et al., 2009). Other studies provided interesting results supporting the importance of acclimatization processes for the cardiorespiratory, muscular and haematological systems (Chapman et al., 1998). A study concluded that individuals chronically residing between 1675 and 2255 m acclimatize more rapidly when exposed to 4056 m compared with sea‐level populations (Muza et al., 2004). Another study compared the acute adaptations to a hypobaric hypoxia exposure (4300 m) of two groups of military personnel: the first group comprised moderate‐altitude residents (≥3 months of residence between 1800 and 2200 m) and the second group was of low‐altitude residents (Staab et al., 2013). The group of moderate‐altitude residents showed better haematological and ventilatory acute adaptations to 4300 m than the low‐altitude group. These better adaptations led to a lower prevalence/severity of acute mountain sickness. In addition, (Fulco et al., 2007) reported that acclimatized active subjects in moderate altitude preserved their performance better in a prolonged maximal effort at 4300 m. However, these studies are of only limited relevance to long‐term residents at low to moderate altitude because: (1) acclimatization periods are restricted to a few months; (2) the focus is on responses to altitudes above 4000 m, which are seldom experienced in competition or training camps; and (3) the participants are not highly trained endurance athletes.

To the best of our knowledge, it is not known whether Caucasian endurance‐trained athletes have better performance at moderate altitude (frequently visited for training and competition) when they live and train for several years at low altitude. To investigate the physiological benefits or to accept that this idea is a myth, because of insufficient hypoxic stimulus, we studied the responses to exercise of two groups of endurance‐trained athletes: one group of low‐altitude resident athletes (Cerdanya) and one group of athletes living at sea level (Barcelona), in their lifetime environment (1200 m or sea level) and at a simulated moderate altitude (2500 m).

## 2 MATERIALS AND METHODS

### 2.1 Ethical approval

Approval for this study was obtained from the institutional ethical committee (Comite de etica del Hospital Germans Trias i Pujol #PI‐17‐115). All experiments were performed in accordance with the *Declaration of Helsinki*, and all athletes signed a consent form.

### 2.2 Subjects

Two groups of male Caucasian athletes were recruited in the present study on the basis of documented residence history, without genetic screening. They were between 18 and 30 years old. The first group (*n* = 13) consisted of athletes living and training in Cerdanya for several years, in low‐altitude conditions [1200 m, altitude resident group (AR)]. The second group (*n* = 12) consisted of sea‐level‐based athletes living and training near Barcelona at sea level [sea‐level resident group (SLR)]. Each participant met the following inclusion criteria: (1) ≥5 years of life and training in their environment group (altitude or sea level); (2) >5 years of training and competition in endurance sport; (3) >8 h of endurance training per week; and (4) no exposure to high altitude during the 3 weeks preceding the study for SLR participants. Athletes who had cardiorespiratory diseases or athletes with a family history of cardiac pathologies were excluded.

### 2.3 Design

Participants performed one maximal incremental test on a cycle ergometer in two conditions: in their habitual environment (Puigcerda, Cerdanya at 1200 m or Barcelona at SL; BASE), and in simulated altitude at 2500 m (inspired $P_{O_{2}}$ = 109 mmHg; 2500). Exercise tests were performed at the same time of day to limit the effect of circadian rhythm. Tests were randomized and they were conducted in single‐blind fashion; at the end of each session, participants were asked informally which condition they believed they had performed, and no participant consistently identified the condition correctly, although this check was not quantified formally. Athletes were not allowed to have any intense activity for 24 h before the test sessions.

### 2.4 Spirometry

Lung volumes and flow–volume dynamics were evaluated using a portable spirometer (Pony Graphic, Cosmed, Rome, Italy). Tests were conducted following American Thoracic Society recommendations (Anon, 1991). After a forced inspiration, participants made a maximal expiration from their total lung capacity to their residual volume. Tests were repeated until dynamics were reproducible at ±5%. The best trial was used to determine the forced vital capacity (FVC), the forced expiratory volume in 1 s (FEV_1_) and the FEV_1_/FVC ratio. Predicted data were determined using the formulas by Quanjer et al. (1993).

### 2.5 Normobaric hypoxia condition

Altitude corresponding to 2500 m (inspired $P_{O_{2}}$ = 109 mmHg) was simulated by adding medical nitrogen with AltiTrainer^®^ (SMTEC S.A., Geneva, Switzerland) to reduce the fraction of inspired O_2_. In each condition, participants were asked to stay for 4 min at rest before starting exercise. This rest time allows gas mixture diffusion and normalizes the hypoxic stimulus.

### 2.6 Maximal exercise test and gas‐exchange measurements

Participants completed a maximal effort test on a cycle ergometer (Racer 9, Kettler, Germany). The maximal incremental test started with a 3 min warm‐up at 60 W. The power was increased by 30 W every minute until exhaustion. Pedalling frequency was set at the participant's self‐preferred cadence, ranging between 70 and 90 rpm. The test was considered to be maximal if at least three of the following four criteria were met: a change in oxygen uptake (${\dot{V}}_{O_{2}}$) of <100 mL with increasing workload; a heart rate within 10% of the age‐predicted maximal value [210 − (0.65 × age) ± 10%] (Nes et al., 2013); a respiratory exchange ratio of >1.1; and inability to maintain the imposed pedalling frequency despite maximal effort and verbal encouragement. Gas‐exchange parameters [${\dot{V}}_{O_{2}}$, carbon dioxide production (${\dot{V}}_{CO_{2}}$) and their ratio (${\dot{V}}_{CO_{2}}/{\dot{V}}_{O_{2}}$)], minute ventilation (${\dot{V}}_{E}$), breathing frequency and tidal volume were measured by a breath‐by‐breath metabolic analyser (Quark CPET, Cosmed, Rome, Italy) and averaged over 15 s intervals for analysis, consistent with standard practice for maximal incremental exercise testing (Robergs et al., 2010). Ventilatory reserve (VR), expressed as a percentage, was calculated by comparing maximal exercise ventilation (${\dot{V}}_{E,\max}$) relative to maximal voluntary ventilation (MVV) estimated mathematically by multiplying the resting FEV_1_ by 35 (Wasserman et al., 1987), as: VR (%) = [(MVV − ${\dot{V}}_{E,\max}$)/MVV] × 100.

The analyser was calibrated according to the manufacturer's instructions by an ambient air calibration, a 3 L syringe and a gas of known O_2_ and CO_2_ concentrations.

### 2.7 Heart rate and $S_{pO_{2}}$

The heart rate was monitored continuously with a chest belt connected to the metabolic analyser (Garmin, Olathe, KS, USA). The $S_{pO_{2}}$ was measured continuously at rest and during tests with a pulse oximeter probe (Nonin Medical Inc., Plymouth, MN, USA) placed on the ear lobe. Participants’ ears were prewarmed by a vasodilating capsaicin cream (Finalgon, Fher, Spain) to avoid poor tissue perfusion. To maintain permanent contact during exercise, the probe was held in place with adhesive tape.

### 2.8 Statistical analysis

Data are expressed as the mean ± SD. Initially, data were assessed for normality using the Shapiro–Wilk test. To compare the characteristics of two populations, Student's unpaired *t*‐test (for parametric data) and a Mann–Whitney unpaired *U*‐test (for non‐parametric) were conducted. A mixed model for repeated measures was applied to assess the main effects of group (SLR vs. AR), condition (BASE vs. 2500) and the interaction of these two variables. Pairwise comparisons evaluated exercise‐related changes. For between‐group comparisons analysed with Student's *t*‐test and with the Mann–Whitney *U*‐test (Table 1), the effect size was expressed as Cohen's *d* and the rank‐biserial correlation, respectively. For the mixed‐model repeated‐measures analyses (Table 2), the effect size was expressed as partial eta‐squared (η^2^_p_) for each effect (group, condition and group × condition interaction). Pearson's and Spearman's coefficients were used to test the relationship between ${\dot{V}}_{O_{2}\max}$ drop and the other outcomes, according to the data distribution. All statistical analyses were completed using SigmaStat software (v.4). Statistical significance was set at *P <* 0.05.

**TABLE 1:** Subject characteristics for sea‐level residents and altitude residents groups.

Parameter	SLR	AR	*P*‐value, effect size
Age, years	23.3 ± 4.5	22.8 ± 2.5	*P =* 0.659, *r* = 0.11
Height, cm	178 ± 5.4	174 ± 3.7	*P =* 0.122, *d* = −0.66
Body mass, kg	66.8 ± 5.3	63.4 ± 5	*P =* 0.049, *r* = 0.47
FVC, %	109 ± 11.5	107 ± 9.5	*P =* 1.00, *r* = −0.01
FVC, L	5.6 ± 07	5.5 ± 0.8	*P =* 0.201, *r* = 0.31
FEV_1_, %	108 ± 13.2	104 ± 9.0	*P =* 0.378, *d* = −0.37
FEV_1_, L	4.8 ± 0.6	4.5 ± 0.6	*P =* 0.265, *r* = 0.27
FEV_1_/FVC, %	84 ± 5.0	83 ± 4.5	*P =* 0.882, *d* = −0.06
Peak expiratory flow, %	101 ± 15.6	101 ± 23.4	*P =* 0.951, *d* = −0.02
Peak expiratory flow, L s^−1^	9.5 ± 2.0	10.0 ± 2.4	*P =* 0.617, *d* = 0.20
Training, h week^−1^	12 ± 2.8	12.5 ± 3.5	*P =* 0.672, *d* = 0.17
Years of practice, years	8.8 ± 3.4	8.5 ± 3.4	*P =* 0.830, *d* = −0.09

*Note*: Values are the mean ± SD.

Abbreviations: AR, altitude residents; FVC, forced vital capacity; FEV_1_, forced expiratory volume in 1 s; SLR, sea‐level residents.

**TABLE 2:** Evolution of maximal exercise parameters for sea‐level residents and altitude residents groups from BASE to 2500 environment.

Parameter	Condition	SLR (*n* = 12)	AR (*n* = 13)	Group	Condition	Group × Condition
HR_max_, beats min^−1^	BASE	181.6 ± 8.9	178.6 ± 12.1	*P* = 0.701	*P* = 0.003	*P* = 0.232
	2500	176.6 ± 8.9	176.4 ± 12.2	η^2^_p_ *=* 0.007	η^2^_p_ *=* 0.311	η^2^_p_ *=* 0.061
$S_{pO_{2}\max}$, %	BASE	94 ± 4.8	93.8 ± 3.1	*P* = 0.006	*P* < 0.001	*P* < 0.001
	2500	82 ± 3.9^***^	91.6 ± 4.6^†††*^	η^2^_p_ *=* 0.287	η^2^_p_ *=* 0.809	η^2^_p_ *=* 0.678
${\dot{V}}_{E,\max}$, L min^−1^	BASE	156.6 ± 25.4	179.2 ± 29.9	*P* = 0.067	*P* = 0.425	*P* = 0.444
	2500	156.5 ± 26.6	174.3 ± 27	η^2^_p_ *=* 0.139	η^2^_p_ *=* 0.030	η^2^_p_ *=* 0.026
VR, %	BASE	6.1 ± 13.6	−13.4 ± 18.5^††^	*P* = 0.004	*P* = 0.413	*P* = 0.624
	2500	6.8 ± 9.6	−10.8 ± 16.6^††^	η^2^_p_ *=* 0.315	η^2^_p_ *=* 0.031	η^2^_p_ *=* 0.011
${\dot{V}}_{O_{2}\max}$, ml min^−1^ kg^−1^	BASE	73.4 ± 6.6	71.9 ± 6.9	*P =* 0.685	*P* < 0.001	*P* = 0.027
	2500	62 ± 6.3^***^	65.5 ± 6.6^***^	η^2^_p_ *=* 0.007	η^2^_p_ *=* 0.752	η^2^_p_ *=* 0.196
Maximal power output, W	BASE	425 ± 46.2	416.5 ± 45	*P =* 0.937	*P* < 0.001	*P =* 0.012
	2500	378.7 ± 39.5^***^	390 ± 46.6^***^	η^2^_p_ *=* 0.000	η^2^_p_ *=* 0.813	η^2^_p_ *=* 0.245

*Note*: Values are the mean ± SD.

Abbreviations: AR, altitude residents; HR_max_, maximal heart rate; SLR, sea‐level residents; SpO_2__max_, Pulse oxygen saturation at maximal exercise; Ve_max_, ventilation at maximal ercise; VO_2max_, maximal oxygen uptake; VR, ventilatory reserve.

Within‐group difference between BASE and 2500: ^*^ *P <* 0.05, ^**^and ^***^ *P <* 0.001.

Between‐group difference in the same condition: ^††^ *P <* 0.01 and ^†††^ *P <* 0.001.

## 3 RESULTS

The anthropometric, spirometry and training‐related characteristics of the two groups are described in Table 1. No significant between‐group difference was reported. The AR group had lived in their environment for 7.5 ± 6.7 years. The SLR group had lived at sea level since birth. At BASE, no significant differences were observed between SLR and AR for resting heart rate or resting $S_{pO_{2}}$, respectively (69.2 ± 11.1 vs. 74 ± 14.8 beats min^−1^) and (99.5% ± 0.5% vs. 99% ± 0.7%).

**FIGURE 1:**
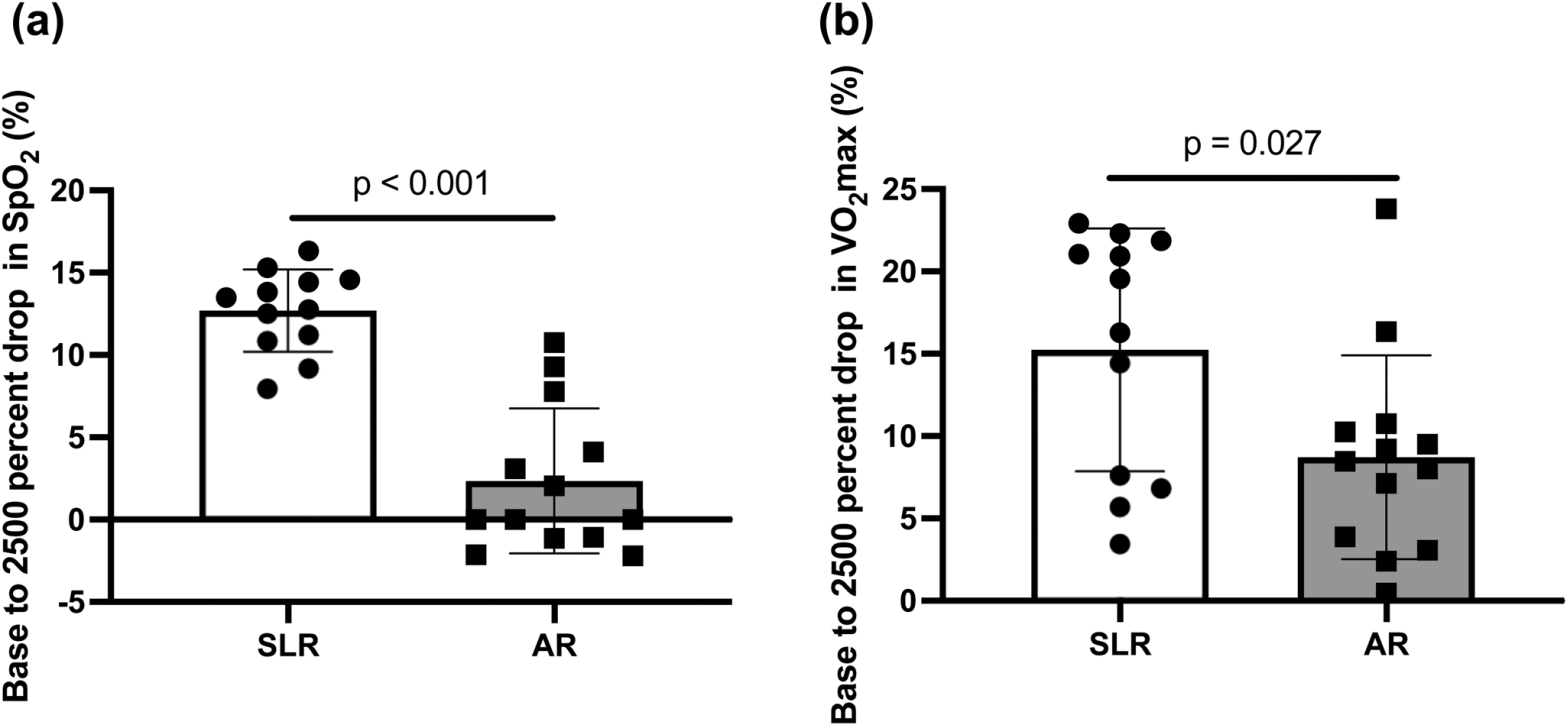
Evolution of maximal oxygen saturation ($S_{pO_{2}\max}$; a) and maximal oxygen uptake (${\dot{V}}_{O_{2}\max}$; b) values between BASE and 2500 environment for athletes who were sea‐level residents (circles; *n* = 12) and altitude residents (squares; *n* = 13) group. Values are the mean ± SD.

All exercise tests were maximal, according to the criteria defined in the Materials and methods. The results are reported in Table 2. The transition from BASE environment to 2500 induced changes in both groups at rest. Heart rate increased in SLR from 69.2 ± 11.1 to 74.5 ± 8 beats min^−1^ and in AR from 74 ± 14.8 to 76.2 ± 17.1 beats min^−1^. There was a significant group × condition interaction for the resting $S_{pO_{2}}$ (*P =* 0.010, η^2^_p_ *=* 0.254), which decreased significantly in the SLR group (99.5% ± 0.5% vs. 96.6% ± 2.1%; *P <* 0.001) but not in the AR group (99.0 ± 0.7% vs. 98.1 ± 1.3%; *P =* 0.112). At maximal exercise, ${\dot{V}}_{O_{2}\max}$, arterial oxygen saturation measured at maximal exercise ($S_{pO_{2}\max}$) and maximal power output were significantly lower at 2500 m compared with the BASE environment in both groups; however, the AR group had significantly higher values of $S_{pO_{2}\max}$ (*P <* 0.001, η^2^_p_ *=* 0.678). In both conditions, there was a trend towards higher ${\dot{V}}_{E,\max}$ in the AR group compared with the SLR group (*P =* 0.067, η^2^_p_ *=* 0.139). VR values reported were lower (*P =* 0.004, η^2^_p_ *=* 0.315) in AR in both conditions, whereas ${\dot{V}}_{E,\max}$ was not affected. The percentage drop in $S_{pO_{2}\max}$ (*P <* 0.001, *d* = 2.875) and ${\dot{V}}_{O_{2}\max}$ (*P =* 0.027, *d* = 0.963) between BASE and 2500 m was smaller in the AR group compared with the SLR group (Figure 1).

**FIGURE 2:**
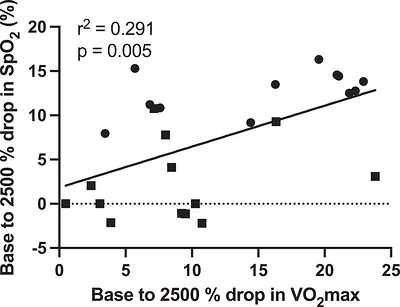
Relationship between drop in maximal oxygen saturation ($S_{pO_{2}\max}$) and drop in maximal oxygen uptake (${\dot{V}}_{O_{2}\max}$) during transition from BASE to 2500, for athletes who were sea‐level residents (circles; *n* = 12) or altitude residents (squares; *n* = 13).

**FIGURE 3:**
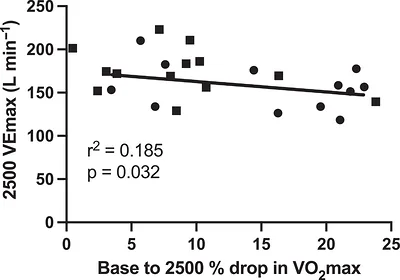
Relationship between maximal minute ventilation (${\dot{V}}_{E,\max}$) at 2500 m and the drop in maximal oxygen uptake (${\dot{V}}_{O_{2}\max}$) during transition from BASE to 2500, for athletes who were sea‐level residents (circles; *n* = 12) and altitude residents (squares; *n* = 13).

A significant correlation (*r* ^2^ = 0.291; *P =* 0.005) was found between the drop in $S_{pO_{2}\max}$ and the drop in ${\dot{V}}_{O_{2}\max}$ observed during the transition from BASE to 2500, with SLR appearing to be the main driver of this relationship (Figure 2). Figure 3 highlights a significant but weak correlation between ${\dot{V}}_{E,\max}$ measured at 2500 m and the decrease in ${\dot{V}}_{O_{2}\max}$ when moving from BASE to 2500 (*r* ^2^ = 0.185; *P =* 0.032), with SLR once again appearing to be the most affected.

## 4 DISCUSSION

In the present study, we compared the physiological responses to exercise at moderate altitude (2500 m) between endurance‐trained athletes permanently residing and training at low altitude (Cerdanya, 1200 m) and their counterparts living and training at sea level (Barcelona). The principal finding is that athletes chronically exposed to low altitude (AR group) demonstrated better preservation of oxygen saturation and a trend to greater ventilatory level during maximal exercise at 2500 m compared with sea‐level residents (SLR group). Importantly, these advantages were associated with significantly greater maintenance of ${\dot{V}}_{O_{2}\max}$.

### 4.1 Comparison with previous studies

Few studies have focused on exposure to low altitude. Even fewer have examined chronic exposure over several years. Studies by Gore et al. (1996, 1997) examined exercise responses in endurance‐trained athletes during acute exposure to a low altitude of 580 m. In these studies, athletes exhibited a significant reduction in arterial O_2_ saturation at 580 m compared with untrained subjects, accompanied by a decrease in ${\dot{V}}_{O_{2}\max}$ closely related to the reduction in arterial oxygen content. Another study reported a significant increase in ventilation at 100 W of exercise at 1560 m, accompanied by a drastic reduction in arterial oxygen saturation in healthy but non‐endurance‐trained participants (Sinkeldam et al., 2014). Thus, even acute exposure to a relatively low altitude can induce measurable physiological responses, which appear to be more pronounced in highly endurance‐trained individuals. Although it has been reported recently that acute responses to hypoxia are influenced by fitness level (Raberin, Manferdelli et al., 2024), to our knowledge no study has specifically evaluated the effects of chronic exposure to low altitude on exercise adaptations according to training status (endurance‐trained athletes vs. untrained individuals). Indeed, the available Swiss studies, particularly those by Burtscher and colleagues, have focused primarily on health outcomes in low‐altitude residents rather than on differential adaptations related to training level (Burtscher et al., 2021).

Given the absence of specific data, it is therefore necessary to draw upon the broader literature on adaptations to moderate altitude. Available evidence indicates that exposure (from a few days to several weeks) to moderate altitude improves ventilatory and haematological responses compared with sea‐level residents. However, these adaptations are often transient and show considerable interindividual variability (Chapman et al., 2016). An additional layer of complexity is introduced by exercise‐induced hypoxaemia (EIH), a phenomenon observed in a large proportion of endurance‐trained athletes (Constantini et al., 2017; Raberin, Manferdelli et al., 2024), which might further modulate certain adaptations to altitude/hypoxia (Raberin et al., 2020, Raberin, Nader, Lopez Ayerbe, Alfonsi et al., 2021, Raberin Nader, Lopez Ayerbe,Mucci et al., 2021). As expected in a cohort of endurance‐trained athletes, a subset of participants exhibited EIH in normoxic conditions. In the SLR group, EIH was classified as mild in two athletes, moderate in two and severe in one (Dempsey & Wagner, 1999). EIH classification was not applicable to the AR group, because their habitual environment (1200 m) is already associated with a reduced ambient $P_{O_{2}}$. In such conditions, the hypoxic stimulus inherent to altitude exposure might confound the distinction between true exercise‐induced desaturation and altitude‐related hypoxaemia, rendering standard EIH criteria physiologically inappropriate in this context.

Our findings demonstrate that endurance‐trained athletes residing long term at 1200 m [an altitude generally classified as ‘low’ according to Bärtsch & Saltin (2008)] exhibited physiologically meaningful advantages when exercising in simulated moderate altitude. Our findings suggest that the altitude threshold required to induce physiological adaptations might be lower than traditionally proposed. Although classical altitude physiology literature generally indicates that substantial hypoxia‐induced adaptations emerge above ∼2000–2500 m (Bärtsch & Saltin, 2008; West, 2012), the present data indicate that lower elevations might still elicit meaningful responses when combined with sustained endurance training. Nevertheless, the magnitude of the benefits observed in the present study remain modest compared with high‐altitude native populations, such as Tibetans, Andeans or Ethiopians, who display distinct genetic characteristics, including higher haemoglobin mass, lower alveolar–arterial gradients and more efficient oxygen utilization (Beall, 2000; Brutsaert, 2008). From a ventilatory perspective, a recent study reported increased thoracic ventilation in high‐altitude populations during walking and running, suggesting that environmental exposure and physical activity influence the form and function of the human thorax (Callison et al., 2022).

### 4.2 Potential mechanisms

We reported a significant association between the reduction in $S_{pO_{2}}$ and the decline in ${\dot{V}}_{O_{2}\max}$ from BASE to 2500 (*r* ^2^ = 0.291, *P =* 0.005), indicating that ∼29% of the variance in the ${\dot{V}}_{O_{2}\max}$ reduction was accounted for by hypoxaemia. Although causality cannot be inferred from this association alone, this association is physiologically consistent with the Fick principle, whereby a reduction in oxygen saturation secondary to the altitude‐induced decrease in arterial oxygen content would limit systemic oxygen delivery and ultimately constrain ${\dot{V}}_{O_{2}\max}$ (Webb et al., 2023). Notably, AR athletes exhibited not only smaller absolute reductions in $S_{pO_{2}}$ but also a more limited percentage decline in both $S_{pO_{2}}$ and ${\dot{V}}_{O_{2}\max}$ during the transition from BASE to 2500 m compared with SLR athletes, further reinforcing the physiological advantage conferred by long‐term residence at low altitude. When examined separately within each group, the association between $S_{pO_{2}}$ reduction and ${\dot{V}}_{O_{2}\max}$ decline did not reach statistical significance (AR: *r* ^2^ = 0.07, *P =* 0.39; SLR: *r* ^2^ = 0.27, *P =* 0.08), probably reflecting insufficient statistical power at the subgroup level. The between‐group differences in $S_{pO_{2}}$ preservation, as established by the mixed‐model analysis, nonetheless support this observation. This pattern might reflect chronic ventilatory and/or haematological adaptations developed through long‐term residence at altitude, potentially attenuating the oxygen desaturation during exercise at 2500 m.

Haematological adjustments have been well reported following hypoxic exposure and altitude training, even at moderate elevations (Frese & Friedmann‐Bette, 2010; Garvican‐Lewis et al., 2015). Furthermore, although increases in haematological parameters are frequently documented, improvements in ${\dot{V}}_{O_{2}\max}$ are not always proportionate in chronically hypoxic individuals (Prommer et al., 2007). Additionally, molecular adaptations in skeletal muscle are reported during hypoxic exposure combined with endurance training (Vogt et al., 2001). These adaptations are mediated, in part, through activation of hypoxia‐inducible factor‐1α, which regulates genes involved in angiogenesis, mitochondrial biogenesis and oxidative metabolism. An upregulation of hypoxia‐inducible pathways and mitochondrial remodelling enhance the efficiency of oxygen utilization despite reduced ambient oxygen availability (Ponsot et al., 2006). However, these adaptations have always been reported at moderate and high altitudes.

The maintenance of high $S_{pO_{2}\max}$ values in AR athletes at BASE and 2500 m suggests an enhanced ventilatory drive. Although the increase in minute ventilation was not statistically significant in AR compared with SLR, the observed trend (*P =* 0.067) suggests it. This observation is consistent with previous work showing that chronic residence at moderate altitude enhances ventilatory acclimatization, thereby improving oxygenation during exercise (Muza et al., 2004). Furthermore, in athletes exposed to altitude training, significant ventilatory adaptations have been reported after several days of exposure, in line with long‐term ventilatory responses to hypoxia in trained individuals (Townsend et al., 2016). However, these studies were conducted at altitudes of >1200 m, which exceed the BASE altitude of the AR group investigated in the present study. The present findings suggest that chronic residence at low altitude might also contribute to ventilatory adaptations, although this observation remains at the level of a statistical trend and should be interpreted with caution.

This is in line with the depletion of ventilatory reserve observed in AR athletes, which suggests that chronic residence at low altitude might induce persistent increases in ventilatory drive, possibly mediated by augmented peripheral chemoreceptor sensitivity and long‐term plasticity of respiratory control centres (Dempsey & Forster, 1982). Long‐term hypoxic exposure has been shown to increase ventilatory sensitivity to hypoxia, reflecting a form of ventilatory acclimatization that persists beyond acute stimuli (e.g., enhanced hypoxic ventilatory response) and involves changes in central and peripheral respiratory control mechanisms (Katayama et al., 2001). Although these adaptations might help to preserve arterial oxygenation during hypoxic exercise, operating near maximal ventilatory capacity increases the work of breathing (Sheel & Romer, 2012). Endurance athletes reach very high minute ventilation at maximal exercise, which can impose mechanical ventilatory constraints (Dempsey et al., 2020). The elevated respiratory muscle work can induce respiratory muscle fatigue and activate the respiratory muscle metaboreflex, increasing sympathetic vasoconstriction in locomotor muscles and thereby limiting limb blood flow and performance (Amann, 2012). In AR athletes, the smaller decline in $S_{pO_{2}}$ suggests improved ventilatory compensation; however, the persistent reduction in ${\dot{V}}_{O_{2}\max}$ from BASE to 2500 indicates that additional mechanisms, potentially including respiratory muscle metaboreflex activation, might contribute to performance limitation.

Importantly, the AR group had been chronically exposed to a low‐altitude environment for 7.5 ± 6.7 years. Some athletes had never left this environment since birth, whereas others met the inclusion criterion of having lived and trained there for ≥5 years. Consequently, several participants not only trained but also grew up in sustained hypoxic conditions. Individuals raised in hypoxia are known to exhibit specific physiological adaptations, including larger lung volumes, enhanced pulmonary diffusing capacity and modulated ventilatory responses, all of which contribute to improved oxygenation at altitude (Koep et al., 2025).

### 4.3 Limitations, strengths and perspectives

Limitations must be acknowledged. First, the relatively small sample size reduces statistical power and might limit generalizability. Second, only male athletes were included, precluding conclusions regarding sex‐specific adaptations. Third, haematological measures, such as total haemoglobin mass and red blood cell volume, were not assessed, restricting interpretation of the underlying mechanisms. Finally, the simulated altitude was limited to 2500 m; future research should explore responses at higher altitudes in order to determine whether the observed physiological patterns persist in more severe hypoxic conditions.

A strength of this study is the use of two well‐matched groups of endurance‐trained athletes, residing long term in different environments, and the application of standardized maximal exercise testing in both normoxic and hypoxic conditions. The single‐blind, randomized design further minimized potential bias.

The present findings have important practical implications for athletes and coaches. Chronic residence and systematic endurance training at relatively low altitudes (∼1200 m) might confer partial protection against performance decrements at moderate altitude. These findings suggest that the altitude threshold required to elicit physiologically meaningful adaptations might be lower than the 1800–2000 m commonly recommended for altitude training camps (Millet et al., 2010), provided that exposure is sustained over several years rather than limited to a short‐term training camp. Future research should extend these findings by incorporating comprehensive assessments of lung diffusing capacity, haematological parameters and peripheral muscular adaptations and by evaluating endurance performance in real‐world competition settings at altitude. Particular attention should also be given to the inclusion of female athletes, who are known to exhibit greater sensitivity to hypoxic exposure (Raberin, Burtscher et al., 2024), in order to characterize potential sex‐specific responses better and improve the ecological validity of altitude research.

## 5 CONCLUSION

In conclusion, endurance‐trained athletes permanently residing at low altitude (1200 m) display an improved systemic oxygenation response to hypoxia and consequently better preserve ${\dot{V}}_{O_{2}\max}$ at 2500 m compared with sea‐level counterparts. These findings support the concept of ‘lifelong acclimatization’ through long‐term low‐altitude residence, even at elevations traditionally considered insufficient to elicit strong physiological adaptations.

## AUTHOR CONTRIBUTIONS

Fabienne Durand and Antoine Raberin conceptualized the protocol. Fabienne Durand, Sergi Brau Villaro and Antoine Raberin performed data acquisition, treatment and analysis. Fabienne Durand was responsible for the first draft of the manuscript. Fabienne Durand, Sergi Brau Villaro and Antoine Raberin interpreted the data. All authors critically revised the manuscript for important intellectual content, approved the final submitted version and agree to be accountable for all aspects of the work in ensuring that questions related to the accuracy or integrity of any part of the work are appropriately investigated and resolved. All persons designated as authors qualify for authorship, and all those who qualify for authorship are listed.

## CONFLICT OF INTEREST

The authors declare no conflicts of interest.

## FUNDING INFORMATION

This study received no external funding.

## GENERATIVE AI STATEMENT

Claude (Anthropic) was used solely for language editing and proofreading of the manuscript text (grammar, spelling, and clarity of English phrasing). It was not used for study design, data analysis, data interpretation or generation of scientific content, and all scientific claims remain the sole responsibility of the authors.

## Data Availability

The data that support the findings of this study are available from the corresponding author upon reasonable request. No public repository was used; sharing is restricted to requests that comply with the ethical approval granted for this study.
